# Data on prevalence and distribution of antimicrobial resistance determinants of *Salmonella enterica* isolates from the formal and informal meat sector

**DOI:** 10.1016/j.dib.2019.103883

**Published:** 2019-04-28

**Authors:** Ishmael Festus Jaja, Chinwe Juliana Iwu

**Affiliations:** a Department of Livestock and Pasture Science, Faculty of Science and Agriculture, University of Fort Hare, Alice, 5700, US; b Department of Global Health, Faculty of Medicine and Health Sciences, Stellenbosch University, Cape Town, 7505, South Africa

**Keywords:** Antimicrobial resistance, Meat, South Africa, Informal sector, Foodborne disease

## Abstract

Foodborne pathogen such *Salmonella enterica* is a leading cause of human gastroenteritis worldwide. The potential to cause more severe and prolonged infection increases when the bacteria harbour resistant gene. In this dataset, *S. enterica* PCR confirmed isolates recovered from the formal (n = 33) and informal (n = 15) meat sector were further tested against 15 antimicrobials and 20 resistance determinants using the disc-diffusion method on Muller-Hinton agar and the genotypic antimicrobial resistance determinants by PCR. In addition, multiple antimicrobial resistance phenotype and the multiple antimicrobial resistance indexes were shown. The data suggest that meat from the formal sector harbour resistance capacity than meat from the informal sector.

**Table:** Specification table

Subject area	*Biology, microbiology, public health*
More specific subject area	*Antimicrobial resistance*
Type of data	*Graph, figure*
How data was acquired	*Bacteria culture (Hektoen Enteric Agar, Merck KGaA, Darmstadt, Germany), PCR detection and confirmation of bacteria (using a thermocycler, Bio-Rad Mycycler, USA), DNA extraction and agar gel electrophoresis (using a transilluminator, Alliance 4.7, UK)*
Data format	*Analyzed*
Experimental factors	*Salmonella enterica obtained from meat in the formal and informal sector was isolated using Hektoen enteric agar. Antimicrobial susceptibility testing was performed using the disc-diffusion method on Muller- Hinton agar plates according to the National Committee for Clinical Laboratory Standards. All data was captured into Microsoft Excel®. Descriptive statistic, eg frequencies and percentages were calculated using SPSS version 24. Bar charts were created using Microsoft Excel®.*
Experimental features	*Swab samples were collected from the formal and informal meat sector. Cultured using standard differential media. DNA extraction was done using the boiling method*[1], [2]*. The detection and confirmation of Salmonella enterica and antimicrobial resistance gene were done using conventional polymerase chain reaction (PCR)*. *Oligonucleotide primers targeting the invA gene (284bp) were used for the PCR.* *The PCR conditions for all products used are stated elsewhere*[1]*. All amplified products were separated on 1.5% agarose gels* and the g*els photographed under UV light in a trans-illuminator (ALLIANCE 4.7 France)*
Data source location	*The University of Fort Hare, Alice, South Africa*
Data accessibility	*All data are presented in this article.*
Related research article	*I.F. Jaja, N.L. Bhembe, E. Green, J. Oguttu, V. Muchenje, Molecular characterisation of antibiotic-resistant Salmonella enterica isolates recovered from meat in South Africa, Acta Trop. 190* (*2019*) [1]

**Table:** 

**Value of the data** • The data on the prevalence of Salmonella enterica will be useful in the further assessment of hygiene management systems (HMS) at abattoir in South Africa. • The antimicrobial resistance data on veterinary non-used drugs suggest the need for intensified research on antibiotic stewardship in public health institutions in the country. Hence this data will be useful in tracking antibiotic use by humans. • The obtained data can assist in foodborne disease epidemiology and in preventing foodborne diseases caused by resistant Salmonella enterica.

## 1 Data

**Fig. 1:**
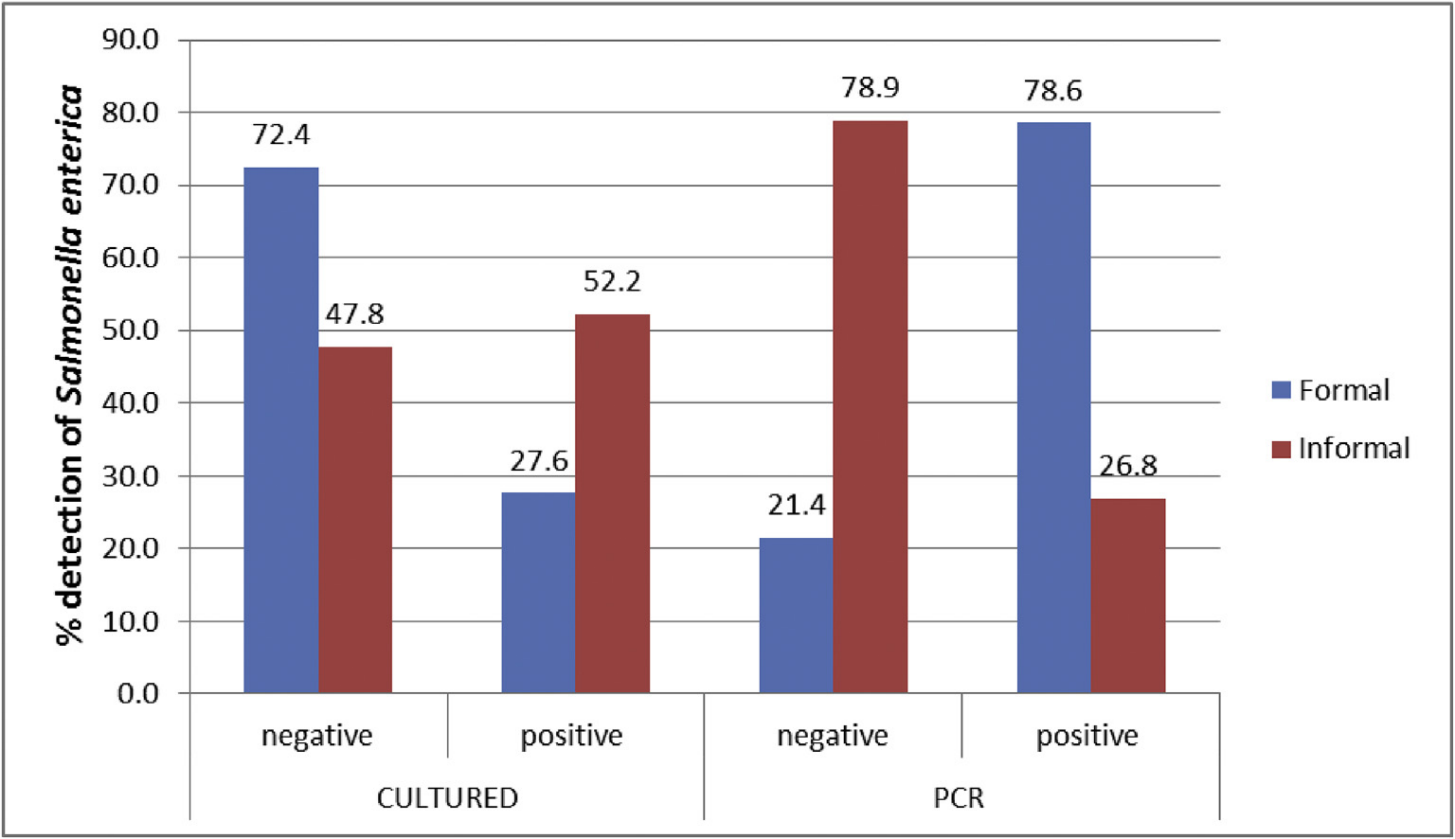
Percentage of *Salmonella enterica* isolated from the formal (n = 152) and informal (n = 136) meat sector.

**Fig. 2:**
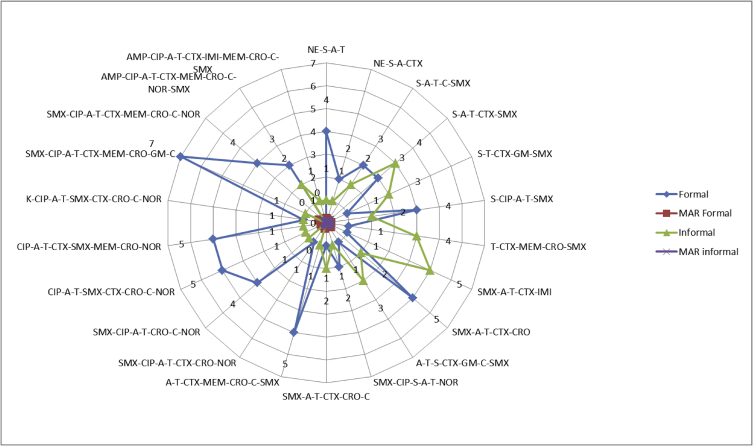
Number and multiple antibiotic resistance (MAR) phenotype in formal and informal sector.

**Fig. 3:**
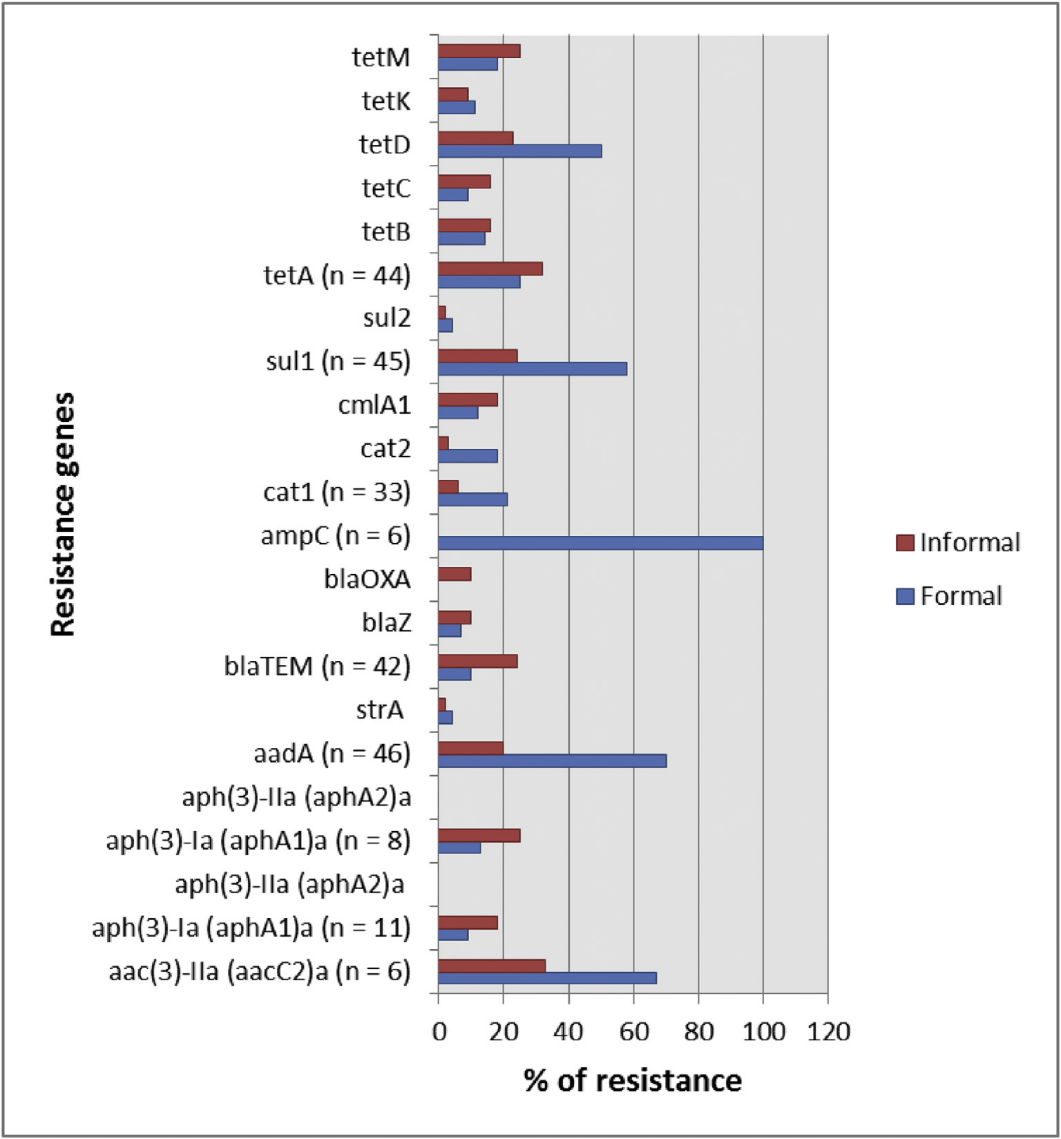
Percentage distributions of antimicrobial resistance determinants among *Salmonella enterica* isolates.

**Fig. 4:**
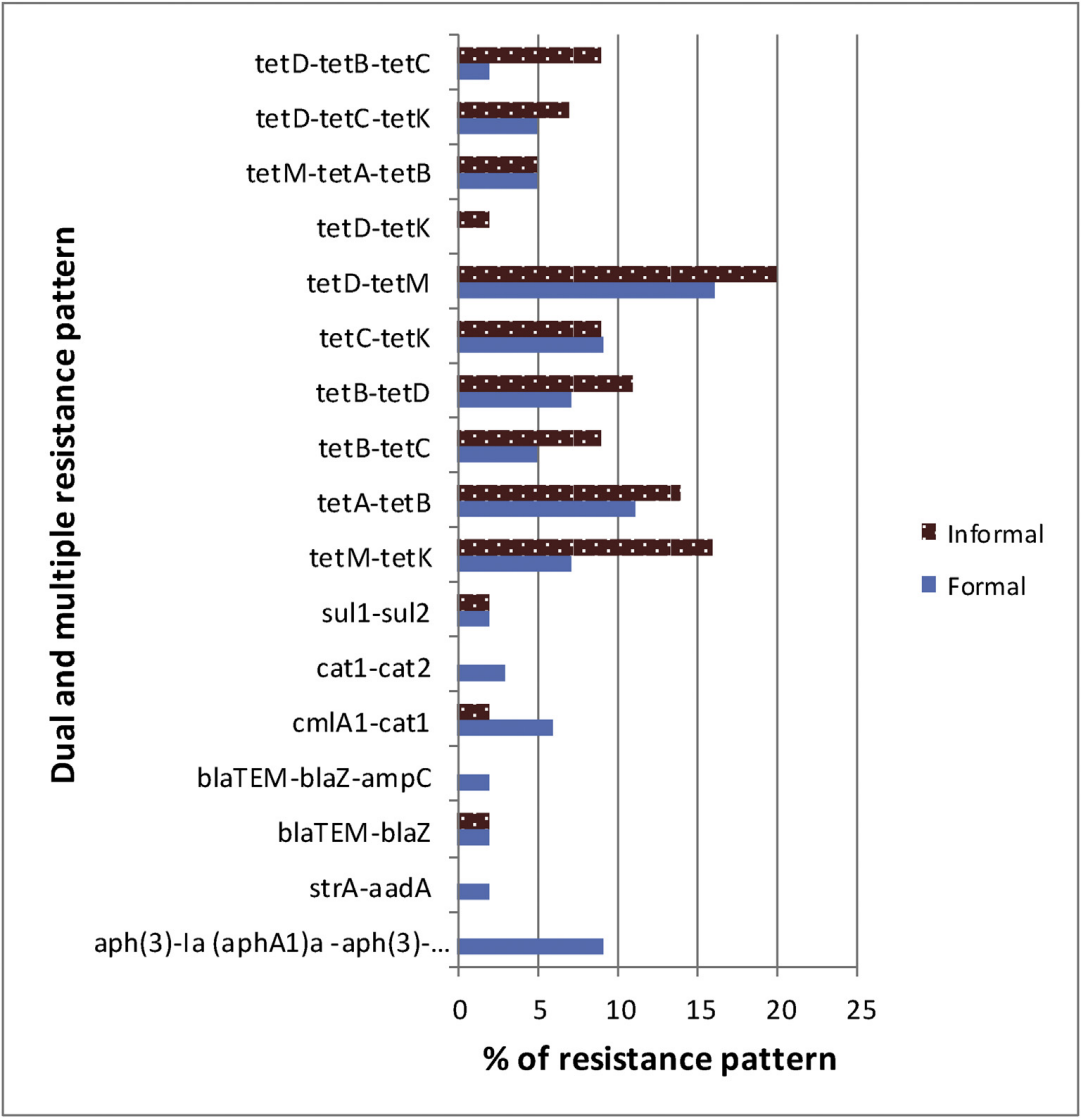
Dual and multiple resistance determinants *Salmonella enterica* isolates.

The presented data show the prevalence and antibiogram of *Salmonella enterica* in the formal and informal meat sector (Fig. 1, Fig. 2). In addition, the data also shows the prevalence of resistance genes and patterns of resistance of the bacteria isolates (Fig. 3, Fig. 4).

## 2 Experimental design, materials, and methods

The swabbing of the animal carcass was done using a sterile cotton throat swab (CTS). The sterile CTS was used on a 100 cm^2^ area on each carcass surface using disposable sterile templates. After the collection of each sample, the throat swabs were placed in individual sterile containers, transported in a cooling box containing ice packs to the Department of Biochemistry and Microbiology, University of Fort Hare laboratory for microbial analysis. Swab samples were subjected to serial dilution for bacteria counting. After the counting, a single distinct colony was taken per sample and stored in glycerol for further use. The stored isolates were resuscitated and streaked on Hektoen enteric agar. Colonies showing characteristic green or blue with a black centre growth on Hektoen Enteric agar medium were selected as Salmonella.

The selected isolate was confirmed using polymerase chain reaction. Confirmed isolates were further screened for their phenotypic antibiotic susceptibility. Criteria for determining whether the isolate is resistant, intermediate or susceptible were adopted from the National Committee for Clinical Laboratory Standards [3]. Phenotypically resistant isolates were selected for genotypic antibiotics resistance testing to 20 resistance genes.
