# Supplementary material for: Data on prevalence and distribution of antimicrobial resistance determinants of Salmonella enterica isolates from the formal and informal meat sector
Source: Data Brief. 2019 Apr 28;24:103883. doi: 10.1016/j.dib.2019.103883 (PMC6520561; doi:10.1016/j.dib.2019.103883)
Supplement: Multimedia component 1 [file mmc1.doc]

**CONFLICT OF INTEREST AND AUTHORSHIP CONFIRMATION**

**PLEASE CHECK THE FOLLOWING AS APPROPRIATE.**

☒ All authors have participated in (a) conception and design, or analysis and interpretation of the data; (b) drafting the article or revising it critically for important intellectual content; and (c) approval of the final version.

☒ The Article I have submitted to the journal for review is original, has been written by the stated authors and has not been published elsewhere.

☒ The Images that I have submitted to the journal for review are original, was taken by the stated authors, and has not been published elsewhere.

☒ This manuscript has not been submitted to, nor is under review at, another journal or other publishing venue.

☒ The authors have no affiliation with any organization with a direct or indirect financial interest in the subject matter discussed in the manuscript

☐ The below authors have affiliations with organizations with direct or indirect financial interest in the subject matter discussed in the manuscript:

Dr. Ishmael Festus Jaja
